# Fecal Microbial Transplantation for the Treatment of Persistent Multidrug-Resistant K*lebsiella pneumoniae* Infection in a Critically Ill Patient

**DOI:** 10.1155/2020/8462659

**Published:** 2020-02-12

**Authors:** V. Ueckermann, E. Hoosien, N. De Villiers, J. Geldenhuys

**Affiliations:** ^1^Department Internal Medicine, University of Pretoria, Pretoria, South Africa; ^2^Department of Clinical Microbiology, Ampath National Reference Laboratory, Centurion, South Africa; ^3^Genetics Department, Ampath National Reference Laboratory, Centurion, South Africa

## Abstract

Dysbiosis of the microbiome is a common finding in critically ill patients, who receive broad-spectrum antibiotics and various forms of organ support. Multidrug-resistant (MDR) organisms are a growing threat in all areas of medicine, but most markedly in the critically ill, where there is both loss of host defences and widespread use of broad spectrum antibiotics. We present a case of a critically ill patient with persistent MDR *Klebsiella pneumoniae* infection, successfully treated with fecal microbiota transplantation (FMT), using stool of a rigorously-screened, healthy donor. FMT for *Clostridium difficile* colitis has been well described in the literature and is an established therapy for recurrent infections with *Clostridium difficile*. The use of FMT for other multidrug-resistant organisms is less frequently described, particularly in the context of critically ill patients. In our case, we have culture-documented clearance of the MDR *Klebsiella pneumoniae* form a patient of FMT.

## 1. Introduction

Dysbiosis (microbial imbalance or maladaptation) of the gut microbiome occurs in the gut of critically ill patients [1]. The role of the gut microbiome in the state of homeostasis, as well as in the pathophysiology of disease has become apparent with the availability of culture-independent methods (such as targeted 16S rRNA Next Generation Sequencing (NGS)) to study the microbiome [2, 3].

Fecal microbiota transplantation is a well-established treatment modality for recurrent *Clostridium difficile* infections and is endorsed by the European Society for Microbiology and Infectious Diseases for this indication [4–7]. There has been widespread interest in the use of FMT in a variety of gastrointestinal and systemic diseases, and several novel indications have emerged in the literature [8].

Here, we present a case of a critically ill patient with severe dysbiosis of his gut microbiome, who underwent FMT with the aim or eradication of a multidrug-resistant organism.

## 2. Case History

A 60-year-old man, with a background history of psoriasis and previous pacemaker insertion (for a dysrhythmia), was admitted to the intensive care unit with a septic shock. The source of infection was the pacemaker wire. He had multiorgan dysfunction manifested as hypotension, acute kidney injury, disseminated intravascular coagulation, and acute respiratory distress syndrome. Blood cultures taken before the initiation of antibiotics revealed a *Klebsiella pneumoniae* which was sensitive to all antibiotics except ampicillin. Source control was attempted by removal of the pacemaker wire and antibiotic therapy with initiation of cefepime . Unfortunately a small part of the wire was attached to the superior vena cava wall and could not be removed without injury to the vessel. Other medical therapy included fluid resuscitation, hemodialysis, mechanical ventilation, thromboprophylaxis, ulcer prophylaxis, and metabolic resuscitation with intravenous thiamine, solucortef, and vitamin C.

After the initial resuscitation efforts, the patient improved and hemodynamic stability was achieved. A white cell localization study revealed activity on the aortic valve, and it was opted to treat the patient as infective endocarditis, with six weeks prolonged course of antibiotic therapy (cefepime). The doses administered were adequate, and prolonged infusions were used to maximize the time above minimum inhibitory concentration.

After two weeks of antibiotic therapy, with the patient liberated from mechanical ventilation and weaned from dialysis, he developed fever, rigors, and an increase in inflammatory markers. Blood cultures were repeated and were once again positive for *Klebsiella pneumoniae*, but now making them resistant to cefepime, and meropenem was initiated. The source of sepsis was actively sought, and it was found that the patient had diverticulitis, which may have been contributory.

Meropenem was continued for four weeks, bringing the total duration of antibiotic therapy to 6 weeks as for infective endocarditis. Antibiotic therapy was discontinued. Within 72 hours of discontinuation of antibiotics, the patient again presented with fever, hypotension, and raised inflammatory markers. Blood cultures were taken, and the *Klebsiella pneumoniae* were cultured once more, this time only sensitive to amikacin, tigecycline, and colistin. A combination of tigecycline and amikacin were initiated with good effect. No new sources of infection could be found.

After fourteen days of therapy and a good clinical response, the antibiotics were stopped. Within days, there was a recurrence of the *Klebsiella pneumoniae*. Throughout the management of this patient, high doses of antibiotics were used, assuming enhanced renal clearance in the critically ill. All sources such as central lines and catheters were removed. After this episode of sepsis was treated, the patient remained stable for a week, after which he developed features of sepsis for the fourth time. Once again, *Klebsiella pneumoniae* was cultured and required the combination of colistin and tigecycline.

Additionally *Candida parapsilosis* was also cultured from blood at this stage. His risk factors for candidemia included the use of numerous broad-spectrum antibiotics, prolonged hospitals stay, indwelling lines, and the use of total parenteral nutrition (TPN). This was successfully treated with an echinocandin.

It became evident the patient had a *Klebsiella pneumoniae* that was suppressed by antibiotics and reappeared once the antibiotics were stopped. Review of the literature revealed case studies in which FMT was used to eradicate resistant microbes [8, 9].

## 3. Methods

### 3.1. Sample Processing and Total Genomic DNA Extraction

To investigate the diversity of the gut microbiome of the patient, a stool sample was taken and stored accordingly at the Ampath National Reference Laboratory for a gut microbiome profiling test. An appropriate amount of the stool sample was suspended in 1 mL of dH20. The sample was centrifuged at 700 ×*g* for 1 minute. A total of 200 *μ*L of the supernatant was used supplemented with 20 *μ*L of Proteinase K (QIAamp DNA mini kit, Qiagen) and 200 *μ*L of ATL Buffer (QIAamp DNA mini kit, Qiagen) for external lysis. The sample was incubated at 56°C for 10 minute followed by a final incubation at 97°C for 15 minutes. Total genomic DNA extraction was performed with a fully automated total nucleic acid extraction and purification protocol on the EMAG® system (BioMérieux). The extracted bacterial genomic DNA was stored at −20°C until further use.

### 3.2. 16S rRNA Sequencing and Analysis

Gut microbiome profiling was performed by utilizing targeted 16S rRNA sequencing with the Ion Torrent 16S™ Metagenomics kit (Thermo Fisher Scientific). The 16S Metagenomics Kit permits polymerase chain reaction (PCR) of 7 hypervariable regions of the 16S rRNA gene. Primer information is exclusive to the design of the kit by Thermo Fisher Scientific. Library preparation was performed according to the Ion 16S Metagenomics kit user guide available online (https://www.thermofisher.com). The E. coli DH10B Control 600 Library was used as positive control from amplification to accurate metagenomic analysis. 16S template preparation was performed on the Ion Chef System (Thermo Fisher Scientific) and sequencing on the Ion S5 XL system (Thermo Fisher Scientific). Sequencing reads were analysed with the Ion 16S™ metagenomic analyses module within the Ion Reporter™ software. For alpha and beta diversity analysis, metagenomic analyses within the Ion Reporter™ software includes Quantitative Insights Into Microbial Ecology (QIIME) algorithms which includes the generation of Krona charts for visualization of microbial communities.

Alpha-diversity calculations generated by the Ion Reporter™ software are based on consensus files generated by the Metagenomics 16S analysis workflow. Operational taxonomic unit (OUT) tables are generated and used for subsequent diversity analysis. Due to the known shortcoming of 16S rRNA sequencing analysis for accurate representation of bacteria up to genus levels, the alpha diversity graphs were based on sequencing reads up to genus level.

Gut microbiome profiling was performed by utilizing targeted 16S rRNA sequencing with the Ion Torrent 16S™ Metagenomics kit (Thermo Fisher Scientific). The 16S Metagenomics Kit permits polymerase chain reaction (PCR) of the hypervariable regions of the 16S rRNA gene. Library preparation was performed according to the Ion 16S Metagenomics kit user guide available online (https://www.thermofisher.com). The 16S template preparation was performed on the Ion Chef System (Thermo Fisher Scientific) and sequencing on the Ion S5 XL system (Thermo Fisher Scientific). Sequencing reads were analysed with the Ion 16S^TM^ metagenomic analyses module within the Ion Reporter™ software.

### 3.3. Fecal Microbial Transplantation

Once severe dysbiosis of the patient's gut microbiome had been proven by a loss of alpha diversity, FMT was considered. The patient's wife was screened as a potential donor, as per the European consensus statement [7]. Meticulous history taking excluded known exposure to HIV, hepatitis, syphilis, Human T-cell lymphotropic virus, or tuberculosis. The donor was in good health with no remote history of gastrointestinal complaints and was not known with any metabolic disorders, autoimmune diseases, or neurological disorders. The donor had no recent travel to tropical countries and no exposure to antibiotics, proton pump inhibitors, and immunosuppressants in the preceding 6 months. A stool sample from the donor was also collected and submitted for gut microbiome profiling as described here previously.

Clinical examination of the donor was normal. Screening blood investigations included cytomegalovirus, Epstein Barr virus, Hepatitis A, B, and C, HIV, Syphilis, full blood count, albumin, C-reactive protein and erythrocyte sedimentation rate, creatinine, electrolytes, aminotransferases, gamma-glutamyltransferase, and alkaline phosphatase. A stool sample was tested for *Clostridium difficile* and enteric pathogens. Occult faecal blood was performed. All the test results were normal. The full battery of investigations performed on the donor is described in supplement A.

Once the donor was deemed suitable, after extensive counselling on the risks of FMT and informed consent for the process, a fecal microbial transplant was performed. One hundred and fifty millilitres of faecal suspension were obtained by blending 30 g of fresh donor stool with 0.9% saline. The faecal preparation was delivered to the patient by a nasojejuneal tube. He was kept at 45 degrees for four hours after the procedure. The procedure was without any complications and was repeated after two weeks.

A second FMT was performed after two weeks, with a fresh stool sample provided by the same donor.

## 4. Results

**Figure 1:**
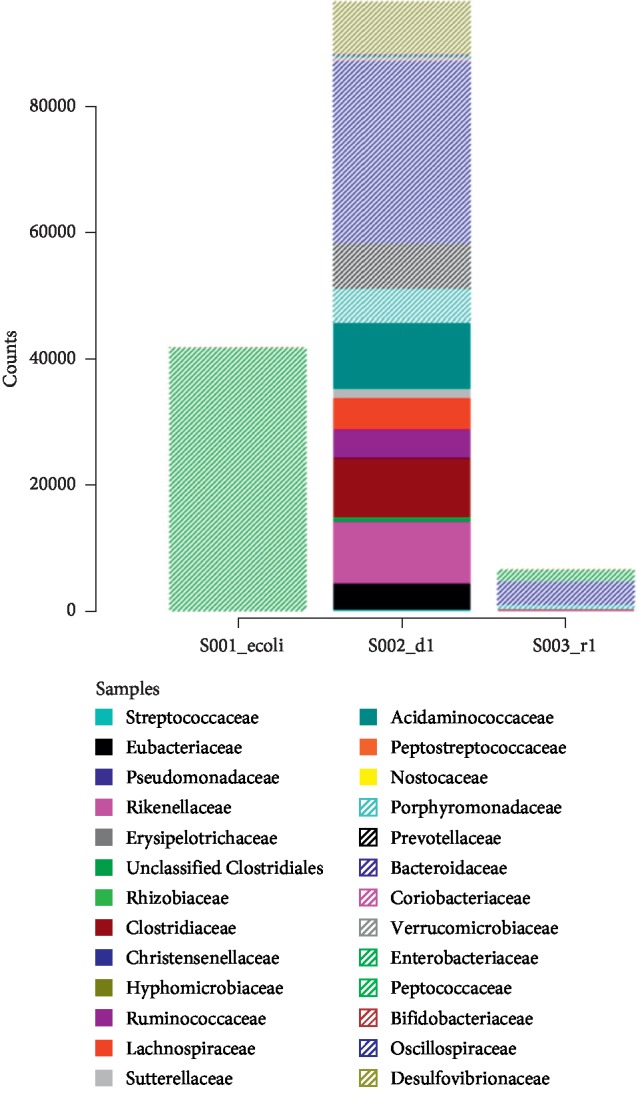
Stacked bar plot indicating the diversity of species in the donor stool (s002_d1) and the recipient (s003_r1). There is an *E. coli* control indicated as s001_ecoli.

16S rRNA sequencing of the gut microbiome of the donor showed a predominance of Bacteroides and Firmicutes. The patient had a higher abundance of Proteobacteria.  Figure 1 is a stacked bar plot generated by the Ion Torrent software, which shows the loss of diversity in the recipient, when compared with the donor, prior to transplant.

**Figure 2:**
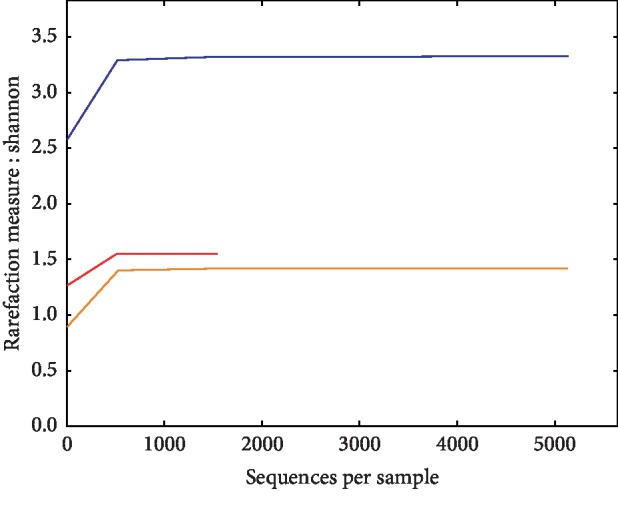
Alpha diversity analysis as illustrated with the Shannon Index indicating the within sample diversity of the patient before and after FMT. The alpha diversity of the donor is illustrated in blue, and that of the patient is illustrated in orange. A control used in the analysis is also indicated in red.

Pretransplant analysis of the stool of both the patient and donor was performed and indicated a lower alpha (within sample) diversity of the patient when compared with the donor (Figure 2). The Shannon index was used to compare the two samples. Typical values for Shannon diversity indices range between 1.5 and 3.5; the higher index is associated with more richness and evenness of microbial communities in the microbiome. In this case, the patient had a Shannon diversity index of less than 1.5, as compared with that of the donor at 3.3. This indicates a loss of microbial diversity in the patient, as can be expected in a critically ill patient who has received antibiotics.

**Figure 3:**
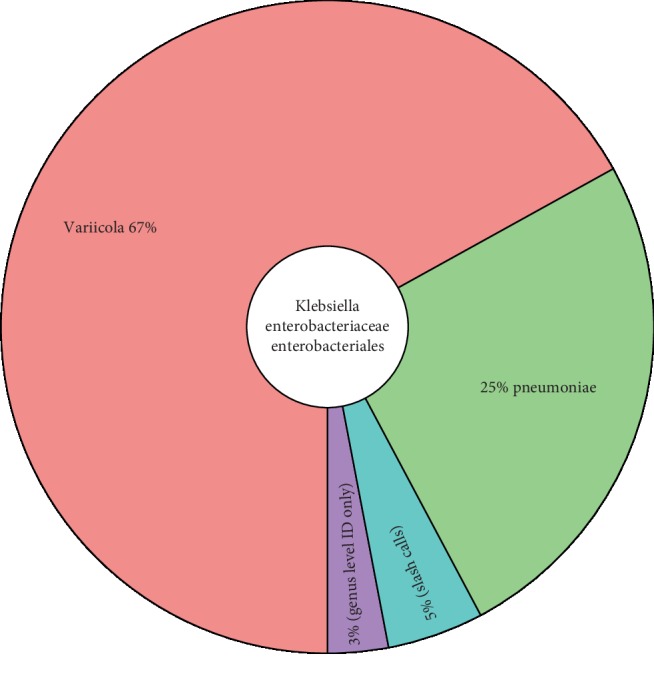
A Krona chart illustrating the identification of *Klebsiella variicola* and *Klebsiella pneumoniae* in the patient prior to FMT.

The intestinal abundance of *Klebsiella* is illustrated in the Krona chart (Figure 3). This abundance of *Klebsiella* supports the notion that the source of the blood stream infection could indeed be the gut, providing further rationale for the FMT.

## 5. Outcome

After the FMT, the patient had no further episodes of sepsis, and blood cultures were repeatedly negative for any bacteria. The patient was successfully discharged from the intensive care unit, and after a period of physical rehabilitation (to address critical illness-associated weakness) returned home in good health.

Six weeks post-transplant stool of the patient was analysed, and the Shannon diversity index had improved to 2, considered within the normal range.

## 6. Discussion

Recurrent *Clostridium difficile infection* is an FDA-approved indication for FMT [1–4]. For this indication, it has proven more effective than medical therapy [6, 10, 11]. There are, however, numerous disease states in which a signal for the benefit of the use of FMT is starting to emerge. Such indications include inflammatory bowel disease and irritable bowel syndrome [8], metabolic syndrome [12], neuropsychiatric diseases [13], haematological diseases [14], and the eradication of resistant microbes [15–22].

When it comes to the use of FMT to eradicate resistant microbes, there are case reports and case series currently found in the literature [15–23]. The organisms for which there are published data include *Escherichia coli*, *Klebsiella pneumoniae*, methicillin-resistant *Staphylococcus aureus*, *Acinetobacter baumannii*, *Pseudomonas aeruginosa*, *and* vancomycin-resistant *Enterococcus*. Seven of eight reported case studies reported eradication of the offending organism at follow-up cultures. There is also a report of reduction in antibiotic inactivation genes on the participant's stool after receiving FMT [17]. The use of FMT in critically ill patients has been reported on less frequently and adds to the novelty of this case. 

Bacteroidetes and Firmicutes phyla are associated with healthy gut microbiomes [24]. Gut microbiome analysis of the donor has indicated a similar result and was therefore considered as a balanced microbiome and used as a reference to the restoration of gut microbial balance of the patient. The donor and the patient shared a number of taxa after FMT, which is expected after FMT and may also refer to the lifestyle and shared environment of the patient and donor. In this clinical case, gut microbiome analysis confirmed the eradication of multidrug-resistant organisms with FMT. Although there was an increase in the Proteobacteria phylum after FMT, restoration of gut microbial balance was indicated by an increase in alpha diversity and an increase in abundance of the Firmicutes phylum.

It is important, in view of the recent literature, to consider the risk of transmitting drug-resistant bacteria during FMT [25]. Traditionally, samples have been screened for carbapenemase-producing enterobacteriacia, but extended-spectrum beta-lactamase production should also be considered.

## 7. Conclusion

We present a case where FMT successfully eradicated multidrug-resistant organisms of the *Klebsiella* genera in a critically ill patient who had suffered recurrent infections with this organism. Further research is needed to define the role of FMT in eradication of multidrug-resistant pathogens in the gut of patients who have been critically ill and to determine the safety of the intervention.
